# Influence of Magnetic Field in Three-Dimensional Flow of Couple Stress Nanofluid over a Nonlinearly Stretching Surface with Convective Condition

**DOI:** 10.1371/journal.pone.0145332

**Published:** 2015-12-29

**Authors:** Tasawar Hayat, Arsalan Aziz, Taseer Muhammad, Bashir Ahmad

**Affiliations:** 1 Department of Mathematics, Quaid-I-Azam University 45320, Islamabad, Pakistan; 2 Nonlinear Analysis and Applied Mathematics (NAAM) Research Group, Faculty of Science, King Abdulaziz University, Jeddah, Saudi Arabia; Tsinghua University, CHINA

## Abstract

This article investigates the magnetohydrodynamic (MHD) three-dimensional flow of couple stress nanofluid subject to the convective boundary condition. Flow is generated due to a nonlinear stretching of the surface in two lateral directions. Temperature and nanoparticles concentration distributions are studied through the Brownian motion and thermophoresis effects. Couple stress fluid is considered electrically conducting through a non-uniform applied magnetic field. Mathematical formulation is developed via boundary layer approach. Nonlinear ordinary differential systems are constructed by employing suitable transformations. The resulting systems have been solved for the convergent series solutions of velocities, temperature and nanoparticles concentration profiles. Graphs are sketched to see the effects of different interesting flow parameters on the temperature and nanoparticles concentration distributions. Numerical values are computed to analyze the values of skin-friction coefficients and Nusselt number.

## Introduction

Boundary layer flow over a continuous stretching surface has various industrial and engineering applications. Such flow commonly involves in the paper production, wire drawing, glass fiber production, extrusion of plastic sheets, hot rolling, cooling of a metallic plate in a cooling bath and many others. Many researchers have discussed the different problems through the linear stretching of the surface but there are various situations in the industrial and technological processes where the stretching of the surface is not necessarily linear. Particularly the flow induced by a nonlinear stretching surface has played important role in the polymer extrusion process. With this viewpoint Vajravelu [1] provided a study to examine the flow and heat transfer properties of viscous fluid induced by a nonlinear stretching surface. Cortell [2] performed a numerical study to investigate the flow of viscous fluid over a nonlinear stretching surface. He studied the two cases of heat transfer namely the constant surface temperature and the prescribed surface temperature. Cortell [3] also explored the flow of viscous fluid over a nonlinearly stretching surface in the presence of viscous dissipation and radiation effects. Hayat et al. [4] addressed the magnetohydrodynamic (MHD) flow over a nonlinear stretching surface by using the modified Adomian decomposition and Padé approximation techniques. Flow and heat transfer properties of nanofluid over a nonlinear stretching surface is reported by Rana and Bhargava [5]. Mukhopadhyay [6] discussed the boundary layer flow over a permeable nonlinear stretching surface subject to partial slip condition. Mabood et al. [7] studied the MHD flow of water-based nanofluid over a nonlinear stretching surface in the presence of viscous dissipation. Recently Mustafa et al. [8] investigated the flow of nanofluid over a nonlinearly stretching surface subject to the convective surface boundary condition.

The insertion of ultrafine nanoparticles (<100nm) in the base liquid is termed as nanofluid. The nanoparticles utilized in nanofluids are basically made of metals (*Cu*, *Al*, *Ag*), oxides (*Al*_2_ *O*_3_) carbides (Si*C*), nitrides (Si*N*, *AlN*) or nonmetals (carbon nanotubes, graphite) and the base liquids like water, oil or ethylene glycol. Addition of nanoparticles in the base liquids greatly enhances the thermal characteristics of the base liquids. Due to such interesting properties, nanofluids are useful in various industrial and technological processes such as the cooling of electronic devices, transformer cooling, vehicle cooling, heat exchanger, nuclear reactor, biomedicine and many others. Especially the magneto nanofluids are useful in MHD power generators, removal of blockage in the arteries, hyperthermia, cancer tumor treatment, magnetic resonance imaging etc. The term nanofluid was first introduced by Choi and Eastman [9] and they illustrated that the thermal properties of base liquids are enhanced when we add up the nanoparticles in it. Boungiorno [10] constructed a mathematical expression to investigate the thermal characteristics of base fluids. Here the effects of thermophoresis and Brownian motion are utilized to enhance the thermal properties of base liquids. Khan and Pop [11] employed the Boungiorno model [10] to analyze the boundary layer flow of nanofluid over a stretching surface. Pujari et al. [12] studied the orientation state of multiwalled carbon nanotubes (MWNTs) dispersions in steady and transient shear flows. Dong and Cao [13] examined the anomalous orientations of rigid carbon nanotube in a sheared fluid. Zhao et al. [14] reported both theoretical and experimental studies of collective effects on the Soret coefficient of particles in deionized (DI) water. Translational thermophoresis and rotational movement of peanut-like colloids under the influence of temperature gradient is addressed by Dong et al. [15]. Wang et al. [16] discussed the thermal diffusion behavior of dilute solutions of very long and thin charged colloidal rods by using the holographic grating technique. Few more recent studies in this direction can be quoted through the investigations [17–27] and several refs. therein.

Most of the studies in the literature explain viscous materials by the classical Navier-Stokes relations. There are several complex rheological materials such as paints, shampoos, slurries, toothpastes, polymer solutions, ketchup, paper pulp, blood, greases, drilling muds, lubricating oils and many others that cannot be characterized through the classical Navier-Stokes expressions. Such materials are known as the non-Newtonian fluids. However, there is no single relation that can present the characteristics of all non-Newtonian fluids. Hence various models of non-Newtonian fluids are developed in the literature. The couple stress fluid model [28–33] is one of such materials. This model has important features due to the presence of body couples, couple stresses and non-symmetric stress tensor. Some interesting examples of the couple stress fluid are blood, suspension fluids, lubricants and electro rheological fluids.

The main aim of the present communication is to generalize the analysis of ref. [8] into three directions. Firstly to consider the three-dimensional flow of couple stress nanofluid. Effects of Brownian motion and thermophoresis are present. We imposed the thermal convective [34,35] and zero nanoparticles mass flux conditions at the surface [36,37]. Secondly to analyze the influence of variable magnetic field under low magnetic Reynolds number assumption. Thirdly to compute the convergent series solutions through the homotopy analysis technique (HAM) [38–45]. Effects of various emerging parameters on the temperature and nanoparticles concentration distributions are sketched and discussed. Skin-friction coefficients and Nusselt number are computed numerically.

## Problem Formulation

Let us consider the steady three-dimensional flow of an incompressible couple stress nanofluid by a bidirectional nonlinear stretching surface. The couple stress fluid is assumed an electrically conducting through a non-uniform magnetic field applied in the *z*−direction. Effects of electric field and Hall current are neglected. The induced magnetic field is not considered subject to the small magnetic Reynolds number. Brownian motion and thermophoresis effects are present. Consider the Cartesian coordinate system in such a way that the *x*− and *y*−axes are taken along the stretched sheet and *z*−axis is perpendicular to it. Let *U*_*w*_(*x*,*y*) = *a*(*x*+*y*)^*n*^ and *V*_*w*_(*x*,*y*) = *b*(*x*+*y*)^*n*^ denote the surface stretching velocities along the *x*− and *y*−directions respectively with *a*, *b*, *n*>0 as the constants. The temperature at the surface is controlled through a convective heating mechanism which is denoted via heat transfer coefficient *h*_*f*_ and temperature of the hot fluid *T*_*f*_ below the surface. The boundary layer expressions governing the flow of couple stress nanofluid are given by

$$\frac{\partial u}{\partial x}+\frac{\partial v}{\partial y}+\frac{\partial w}{\partial z}=0,$$ (1)

$$u\frac{\partial u}{\partial x}+v\frac{\partial u}{\partial y}+w\frac{\partial u}{\partial z}=\nu \frac{\partial^{2}u}{\partial z^{2}}-\nu \prime \frac{\partial^{4}u}{\partial z^{4}}-\frac{\sigma B^{2}\left(x,y\right)}{\rho_{f}}u,$$ (2)

$$u\frac{\partial v}{\partial x}+v\frac{\partial v}{\partial y}+w\frac{\partial v}{\partial z}=\nu \frac{\partial^{2}v}{\partial z^{2}}-\nu \prime \frac{\partial^{4}v}{\partial z^{4}}-\frac{\sigma B^{2}\left(x,y\right)}{\rho_{f}}v,$$ (3)

$$u\frac{\partial T}{\partial x}+v\frac{\partial T}{\partial y}+w\frac{\partial T}{\partial z}=\alpha \frac{\partial^{2}T}{\partial z^{2}}+\frac{{\left(\rho c\right)}_{p}}{{\left(\rho c\right)}_{f}}\left(D_{B}\left(\frac{\partial T}{\partial z}\frac{\partial C}{\partial z}\right)+\frac{D_{T}}{T_{\infty }}{\left(\frac{\partial T}{\partial z}\right)}^{2}\right),$$ (4)

$$u\frac{\partial C}{\partial x}+v\frac{\partial C}{\partial y}+w\frac{\partial C}{\partial z}=D_{B}\left(\frac{\partial^{2}C}{\partial z^{2}}\right)+\frac{D_{T}}{T_{\infty }}\left(\frac{\partial^{2}T}{\partial z^{2}}\right).$$ (5)

The subjected boundary conditions are

$$u=U_{w}\left(x,y\right),\;v=V_{w}\left(x,y\right),\;w=0,\;-k\frac{\partial T}{\partial z}=h_{f}\left(T_{f}-T\right),\;D_{B}\frac{\partial C}{\partial z}+\frac{D_{T}}{T_{\infty }}\frac{\partial T}{\partial z}=0\;\text{at }z=0,$$ (6)

$$u\to 0,\;v\to 0,\;T\to T_{\infty },\;C\to C_{\infty }\;\text{as }z\to \infty .$$ (7)

Note that *u*, *v* and *w* are the velocity components in the *x*−, *y*− and *z*−directions respectively, *v* (= *μ*/*ρ*_*f*_) represents the kinematic viscosity, *μ* stands for dynamic viscosity, *ρ*_*f*_ represents the density of base fluid, *v*′ (= n*/*ρ*_*f*_) denotes the couple stress viscosity, n* stands for couple stress viscosity parameter, *σ* denotes the electrical conductivity, $B\left(x,y\right)=B_{0}{\left(x+y\right)}^{\frac{n-1}{2}}$ stands for non-uniform magnetic field, *T* denotes the temperature, *α* = *k/*(*ρc*)_*f*_ represents the thermal diffusivity of fluid, *k* stands for thermal conductivity of fluid, (*ρc*)_*f*_ represents the heat capacity of fluid, (*ρc*)_*p*_ denotes the effective heat capacity of nanoparticles, *D*_*B*_ stands for Brownian diffusion coefficient, *C* denotes the nanoparticles concentration, *D*_*T*_ represents the thermophoretic diffusion coefficient, $h_{f}=h{\left(x+y\right)}^{\frac{n-1}{2}}$ denotes the non-uniform heat transfer coefficient, *T*_∞_ represents the temperature far away from the surface and *C*_∞_ represents the nanoparticles concentration far away from the surface. We now use the following transformations

$$\begin{array}{c} u=a{\left(x+y\right)}^{n}f^{\prime }\left(\eta \right),\;v=a{\left(x+y\right)}^{n}g^{\prime }\left(\eta \right),\\ w=-{\left(\frac{a\nu \left(n+1\right)}{2}\right)}^{\frac{1}{2}}{\left(x+y\right)}^{\frac{n-1}{2}}\left\{\left(f+g\right)+\frac{n-1}{n+1}\eta \left(f^{\prime }+g^{\prime }\right)\right\},\\ \theta \left(\eta \right)=\frac{T-T_{\infty }}{T_{f}-T_{\infty }},\;\phi \left(\eta \right)=\frac{C-C_{\infty }}{C_{\infty }},\;\eta ={\left(\frac{a\left(n+1\right)}{2\nu }\right)}^{\frac{1}{2}}{\left(x+y\right)}^{\frac{n-1}{2}}z. \end{array}$$ (8)

Eq (1) is now satisfied and Eqs (2)–(7) have the following forms

$$f^{\prime \prime \prime }+\left(f+g\right)f^{\prime \prime }-\frac{2n}{n+1}\left(f^{\prime }+g^{\prime }\right)f^{\prime }-Kf^{\left(v\right)}-M^{2}f^{\prime }=0,$$ (9)

$$g^{\prime \prime \prime }+\left(f+g\right)g^{\prime \prime }-\frac{2n}{n+1}\left(f^{\prime }+g^{\prime }\right)g^{\prime }-Kg^{\left(v\right)}-M^{2}g^{\prime }=0,$$ (10)

$$\theta^{\prime \prime }+Pr\left(\left(f+g\right)\theta^{\prime }+Nb\theta^{\prime }\phi^{\prime }+Nt{\theta^{\prime }}^{2}\right)=0,\;$$ (11)

$$\phi^{\prime \prime }+LePr\left(f+g\right)\phi^{\prime }+\frac{Nt}{Nb}\theta^{\prime \prime }=0,$$ (12)

$$f\left(0\right)=g\left(0\right)=0,\;f^{\prime }\left(0\right)=1,\;g^{\prime }\left(0\right)=c,\;\theta^{\prime }\left(0\right)=-\gamma \left(1-\theta \left(0\right)\right),\;Nb\phi^{\prime }\left(0\right)+Nt\theta^{\prime }\left(0\right)=0,$$ (13)

$$f^{\prime }\left(\infty \right)\to 0,\;g^{\prime }\left(\infty \right)\to 0,\;\theta \left(\infty \right)\to 0,\;\phi \left(\infty \right)\to 0.$$ (14)

In above expressions *K* denotes the couple stress parameter, *M* represents the magnetic number, *c* stands for ratio parameter, *Pr* denotes the Prandtl number, *Nb* represents the Brownian motion parameter, *Nt* stands for thermophoresis parameter, *γ* denotes the Biot number, *Le* stands for Lewis number and prime denotes the differentiation with respect to *η*. These variables are defined by

$$\begin{array}{c} K=\frac{\left(n+1\right)\nu^{\prime }a}{2\nu^{2}}{\left(x+y\right)}^{n-1},\;M^{2}=\frac{2\sigma B_{0}^{2}}{a\rho_{f}\left(n+1\right)},\;c=\frac{b}{a},\;Pr=\frac{\nu }{\alpha },\\ Nb=\frac{{\left(\rho c\right)}_{p}D_{B}C_{\infty }}{{\left(\rho c\right)}_{f}\nu },\;Nt=\frac{{\left(\rho c\right)}_{p}D_{T}\left(T_{f}-T_{\infty }\right)}{{\left(\rho c\right)}_{f}\nu T_{\infty }},\;\gamma =\frac{h_{f}}{k}\sqrt{\frac{\nu }{a}},\;Le=\frac{\alpha }{D_{B}}. \end{array}$$ (15)

Skin-friction coefficients and local Nusselt number are given by

$$\begin{array}{c} Re_{x}^{1/2}C_{fx}={\left(\frac{n+1}{2}\right)}^{1/2}\left(f^{\prime \prime }\left(0\right)-Kf^{iv}\left(0\right)\right),\\ Re_{y}^{1/2}C_{fy}=c^{-3/2}{\left(\frac{n+1}{2}\right)}^{1/2}\left(g^{\prime \prime }\left(0\right)-Kg^{iv}\left(0\right)\right),\\ Re_{x}^{-1/2}Nu_{x}=-{\left(\frac{n+1}{2}\right)}^{1/2}\theta^{\prime }\left(0\right). \end{array}$$ (16)

It is seen that the dimensionless mass flux denoted by a Sherwood number *Sh*_*x*_ is now identically zero and *Re*_*x*_ = *U*_*w*_(*x*+*y*)/*v* and *Re*_*y*_ = *V*_*w*_(*x*+*y*)/*v* represent the local Reynolds numbers.

## Series Solutions

Our purpose now is to compute the series solutions via homotopy analysis technique (HAM). The appropriate initial guesses (*f*_0_,*g*_0_,*θ*_0_,*ϕ*_0_) and the corresponding auxiliary linear operators (**L**_*f*_,**L**_*g*_,**L**_*θ*_,**L**_*ϕ*_) for homotopic solutions are selected as follows:

$$f_{0}\left(\eta \right)=1-e^{-\eta },\;g_{0}\left(\eta \right)=c\left(1-e^{-\eta }\right),\;\theta_{0}\left(\eta \right)=\frac{\gamma }{1+\gamma }e^{-\eta },\;\phi_{0}\left(\eta \right)=-\frac{\gamma }{1+\gamma }\frac{Nt}{Nb}e^{-\eta },$$ (17)

$$L_{f}=f^{\prime \prime \prime }-f^{\prime },L_{g}=g^{\prime \prime \prime }-g^{\prime },\;L_{\theta }=\theta^{\prime \prime }-\theta ,L_{\phi }=\phi^{\prime \prime }-\phi .$$ (18)

The above operators have the following properties

$$\begin{array}{l} L_{f}\left[B_{1}+B_{2}e^{\eta }+B_{3}e^{-\eta }\right]=0,\;L_{g}\left[B_{4}+B_{5}e^{\eta }+B_{6}e^{-\eta }\right]=0,\\ L_{\theta }\left[B_{7}e^{\eta }+B_{8}e^{-\eta }\right]=0,\;L_{\phi }\left[B_{9}e^{\eta }+B_{10}e^{-\eta }\right]=0. \end{array}$$ (19)

in which *B*_*i*_ (*i* = 1 − 10) depict the arbitrary constants.

## Convergence Analysis

**Fig 1:**
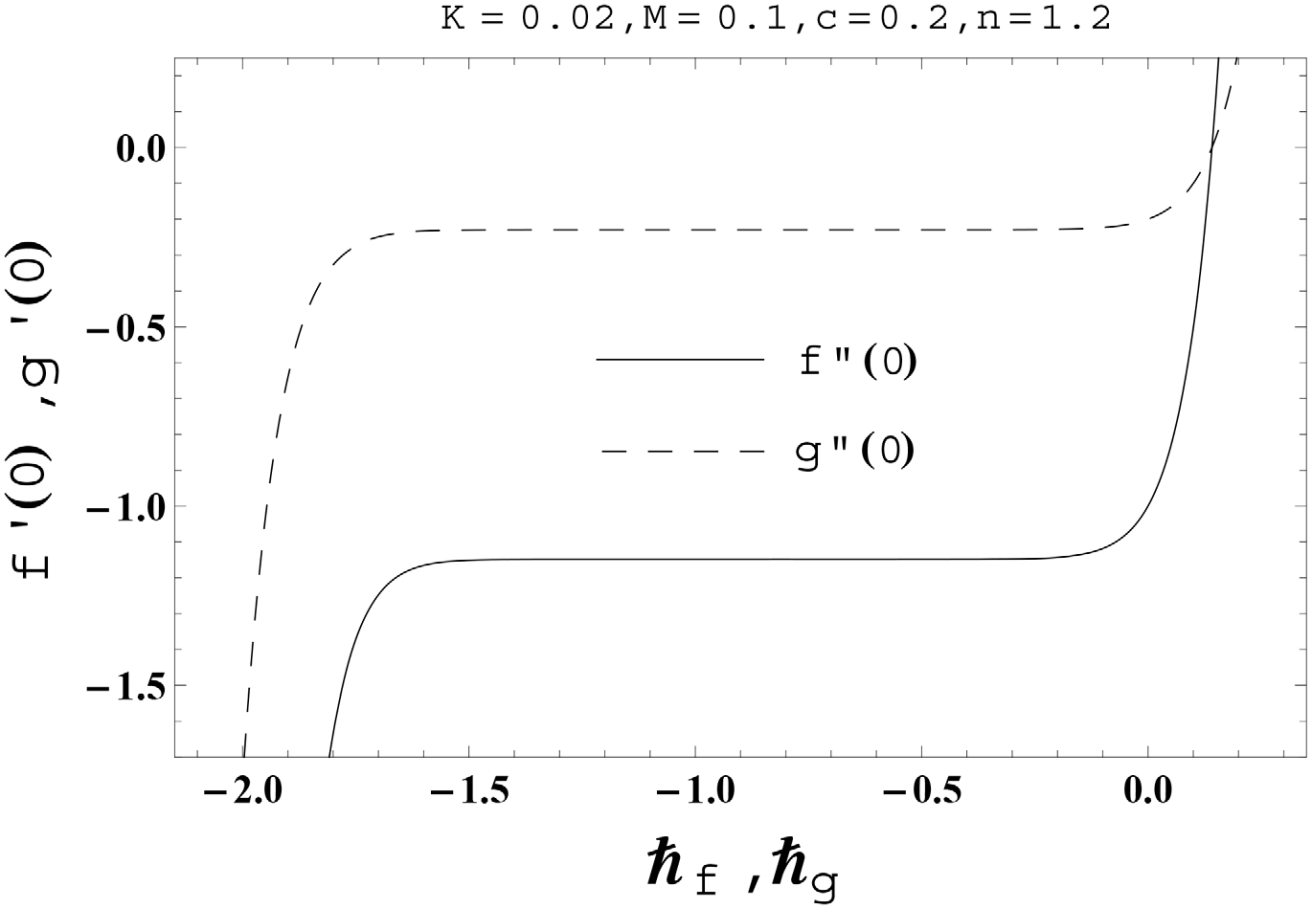
The *ħ*-curves for the functions *f*(*η*) and *g*(*η*).

**Fig 2:**
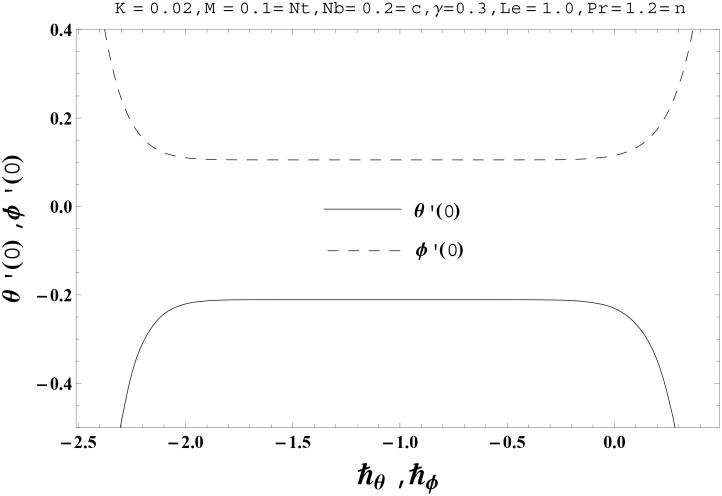
The *ħ*-curves for the functions *θ*(*η*) and *ϕ*(*η*).

No doubt the auxiliary parameters in the series solutions have key role regarding convergence. The proper values of these parameters play a key role to develop the convergent series solutions. For such interest, the *ħ*- curves for the velocities, temperature and nanoparticles concentration profiles are plotted at 15th order of deformations. Figs 1 and 2 clearly indicate that the interval of convergence for *f*, *g*, *θ* and *ϕ* are [-1.50, -0.15], [-1.50, -0.20], [-1.75, -0.15] and [-1.80, -0.20] respectively. Table 1 presents that the 10th order of deformations is enough for the series solutions of velocities, temperature and nanoparticles concentration profiles.

**Table 1:** Convergence of series solutions for various order of approximations when *K* = 0.02, *M* = 0.1 = *Nt*, *c* = 0.2 = *Nb*, *γ* = 0.3, *Le* = 1.0 and *Pr* = 1.2 = *n*.

Order of approximations	-*f*''(0)	-*g''*(0)	-*θ'*(0)	*ϕ'*(0)
1	1.15136	0.23027	0.21692	0.10846
5	1.14888	0.22978	0.21084	0.10542
10	1.14887	0.22977	0.21078	0.10539
20	1.14887	0.22977	0.21078	0.10539
35	1.14887	0.22977	0.21078	0.10539
50	1.14887	0.22977	0.21078	0.10539

## Results and Discussion

**Fig 3:**
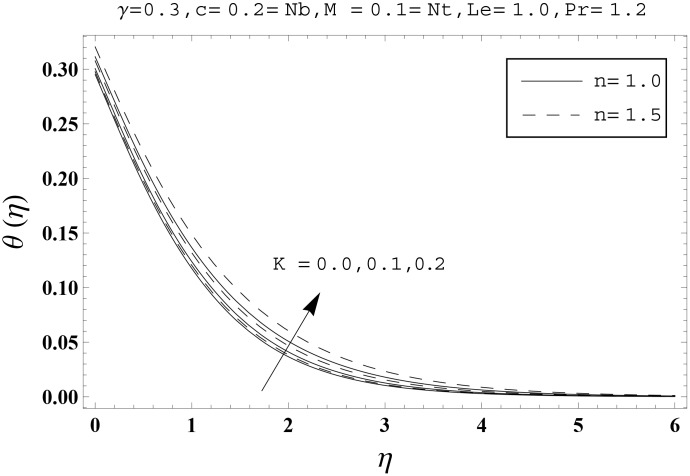
Effects of *n* and *K* on *θ*(*η*).

**Fig 4:**
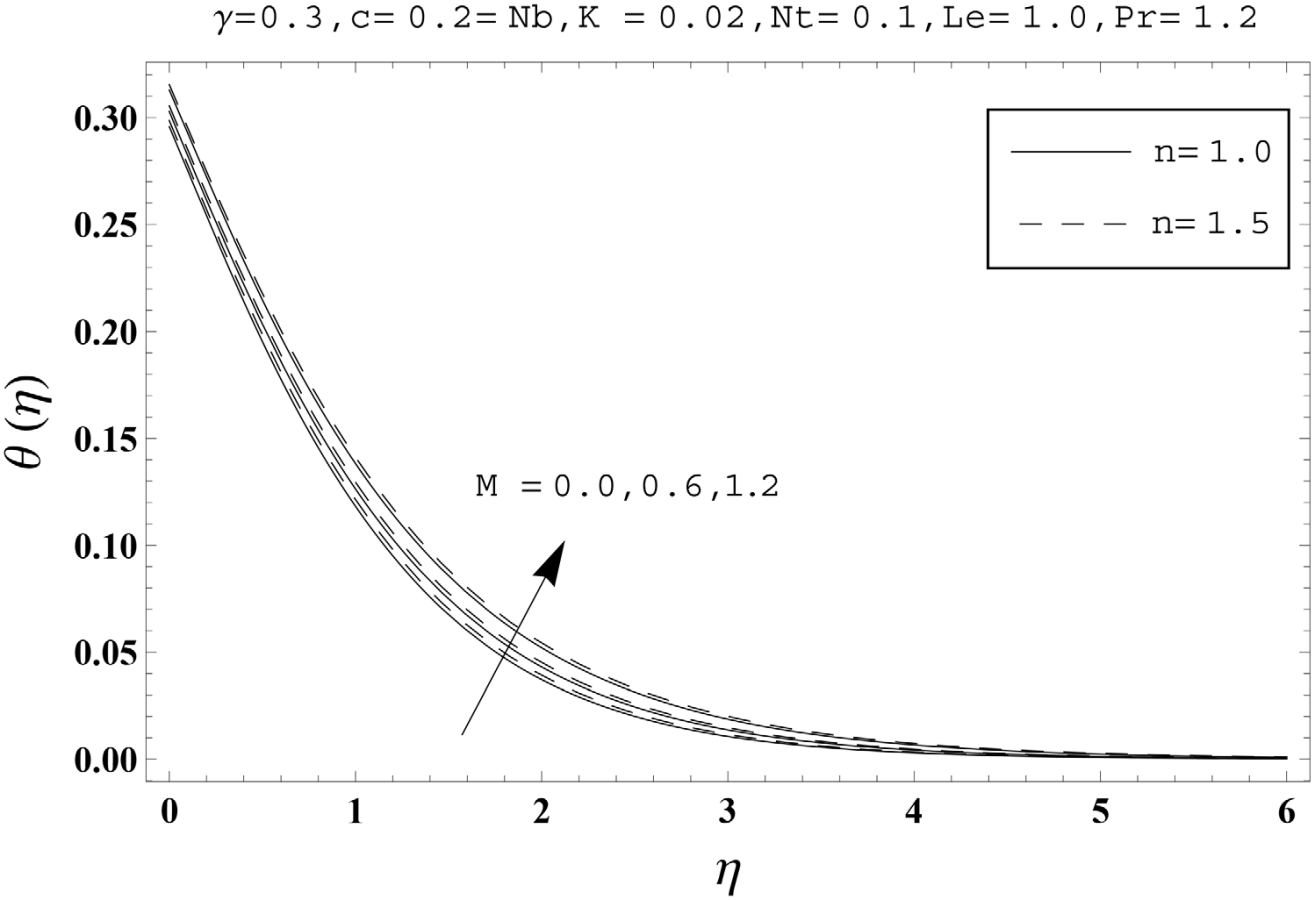
Effects of *n* and *M* on *θ*(*η*).

**Fig 5:**
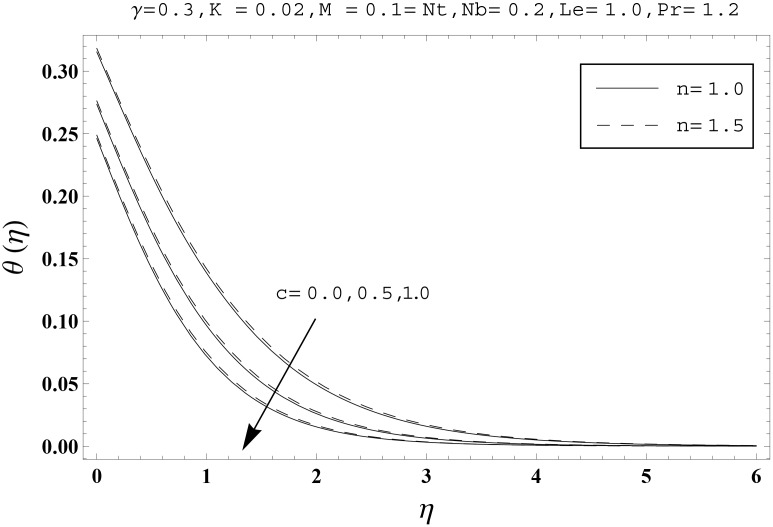
Effects of *n* and *c* on *θ*(*η*).

**Fig 6:**
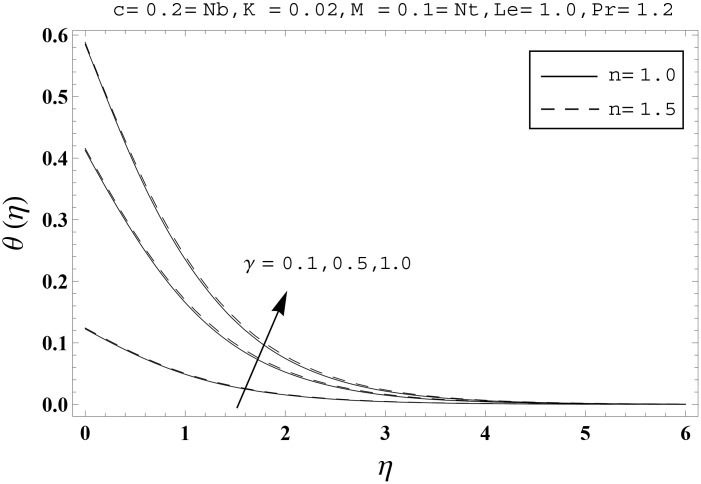
Effects of *n* and *γ* on *θ*(*η*).

**Fig 7:**
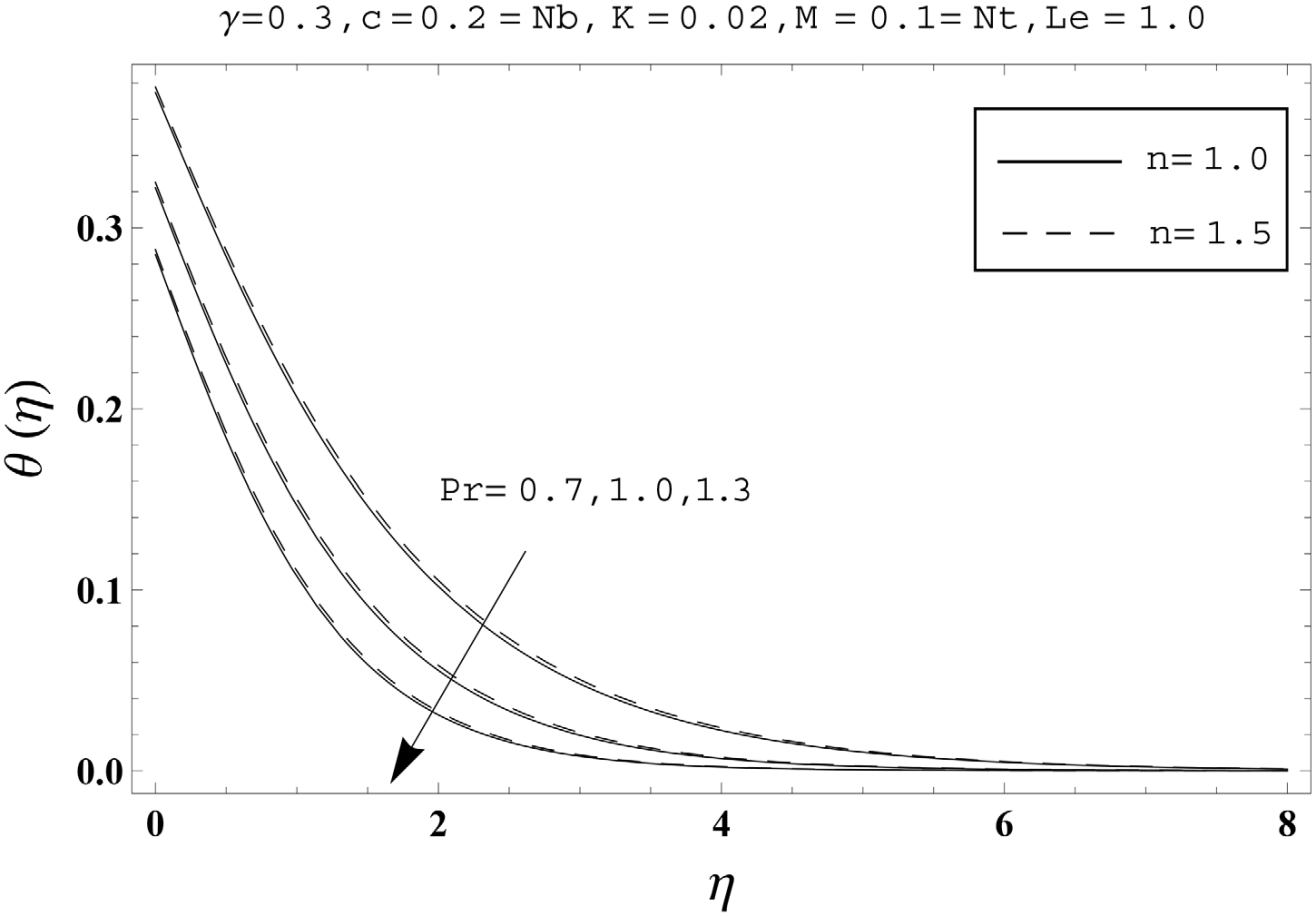
Effects of *n* and *Pr* on *θ*(*η*).

**Fig 8:**
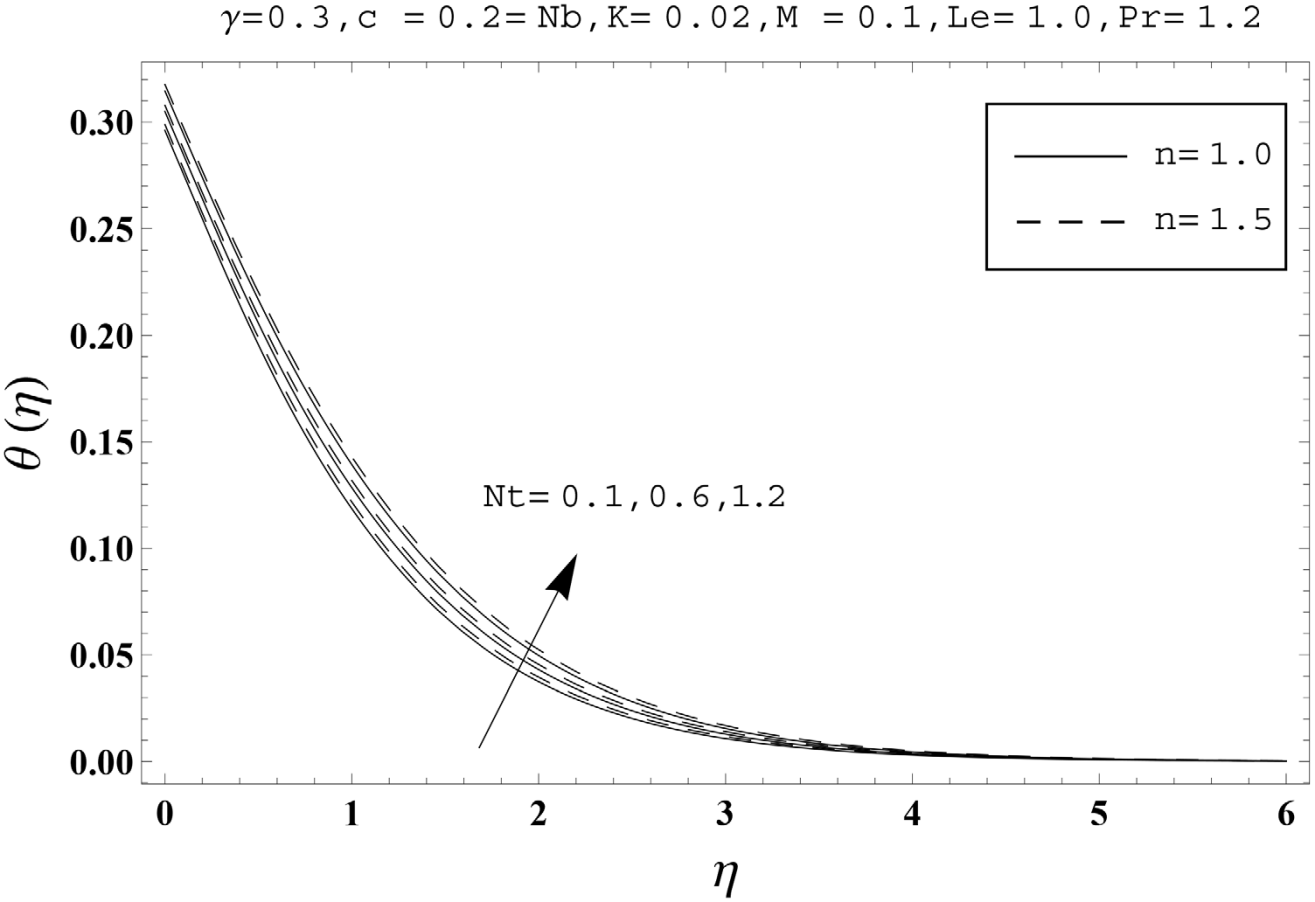
Effects of *n* and *Nt* on *θ*(*η*).

**Fig 9:**
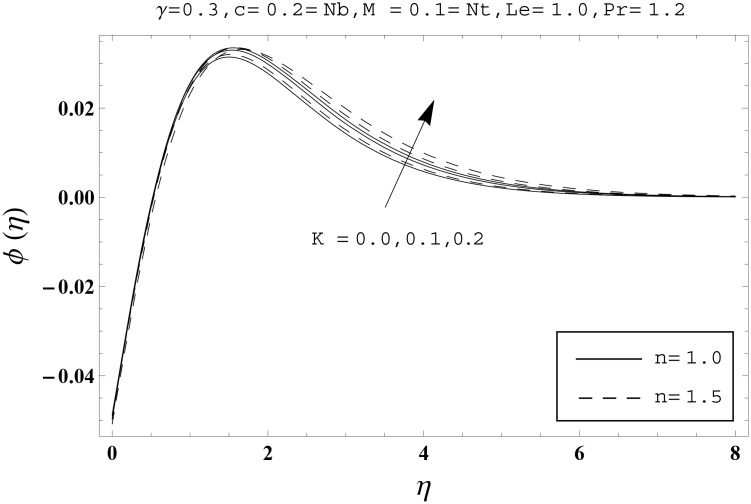
Effects of *n* and *K* on *ϕ*(*η*).

**Fig 10:**
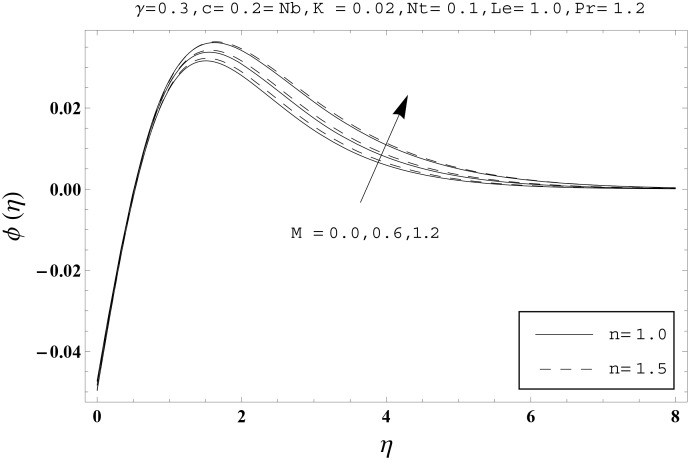
Effects of *n* and *M* on *ϕ*(*η*).

**Fig 11:**
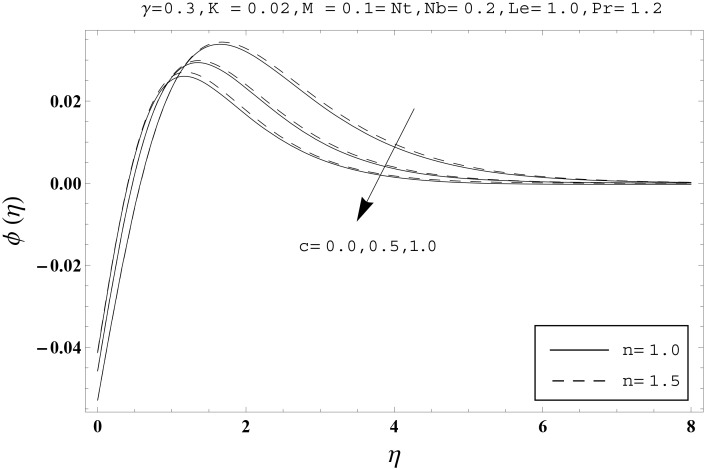
Effects of *n* and *c* on *ϕ*(*η*).

**Fig 12:**
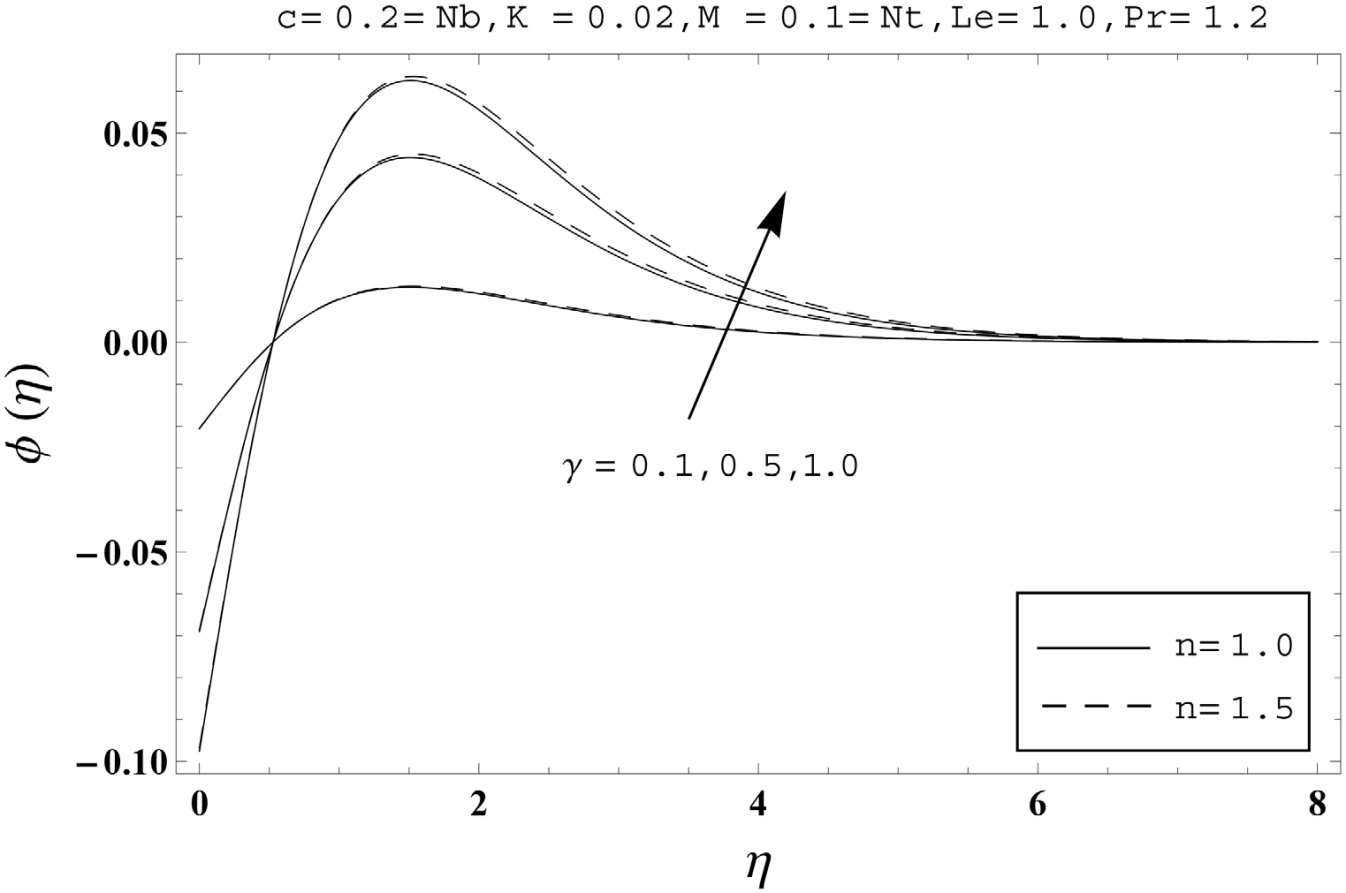
Effects of *n* and *γ* on *ϕ*(*η*).

**Fig 13:**
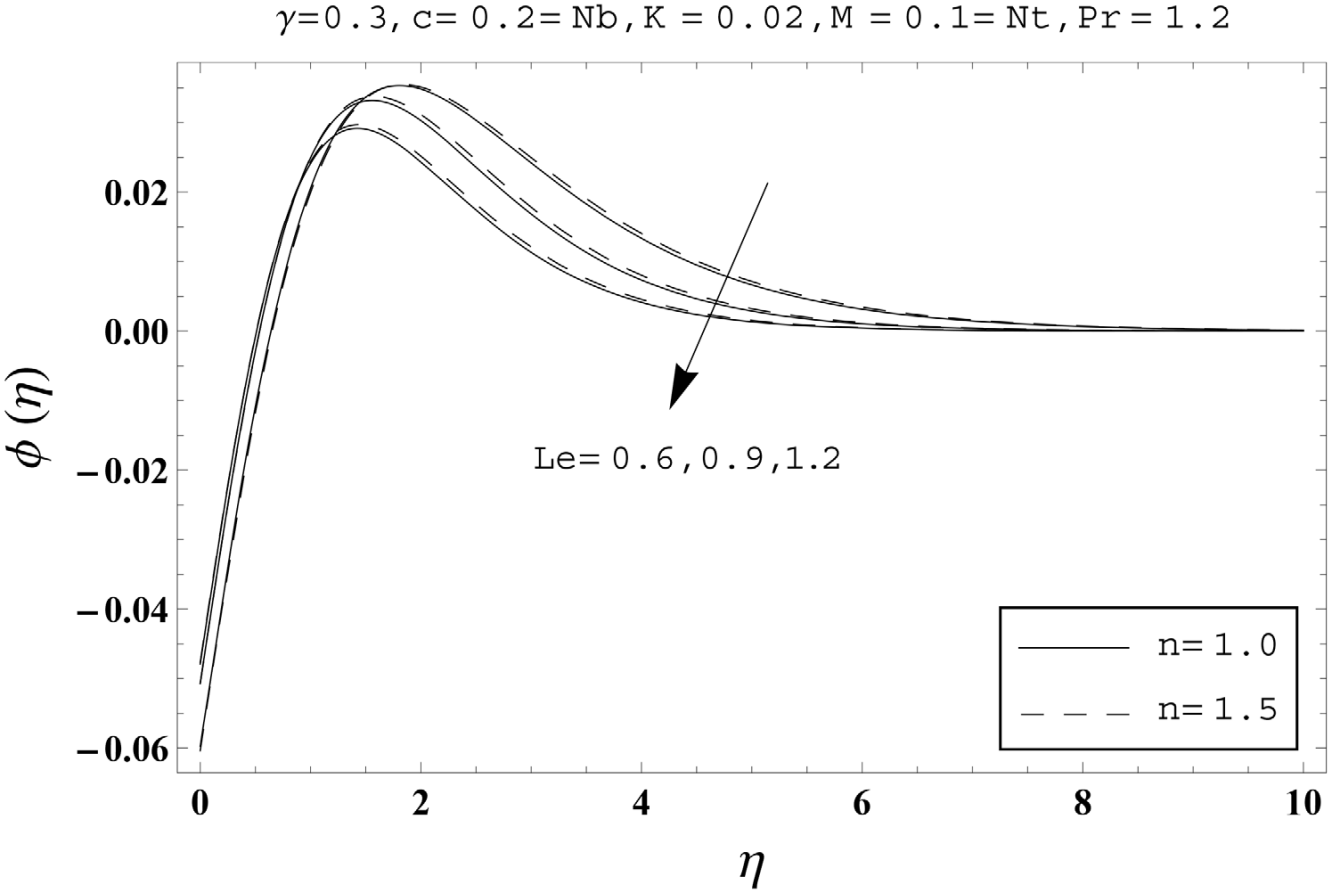
Effects of *n* and *Le* on *ϕ*(*η*).

**Fig 14:**
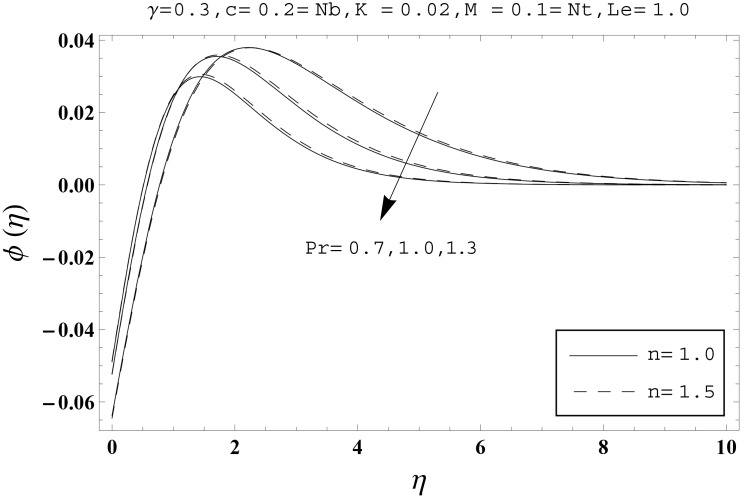
Effects of *n* and *Pr* on *ϕ*(*η*).

**Fig 15:**
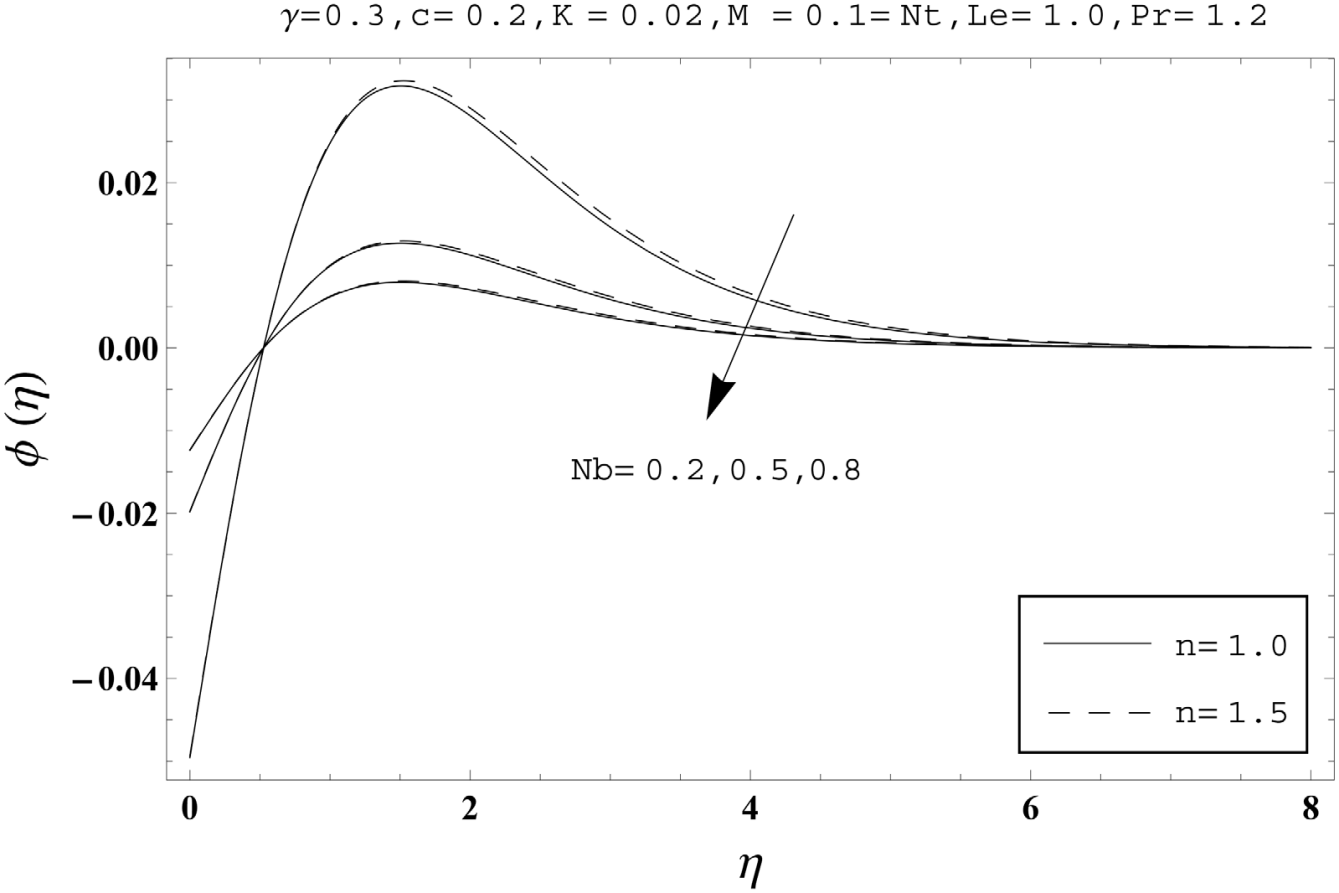
Effects of *n* and *Nb* on *ϕ*(*η*).

**Fig 16:**
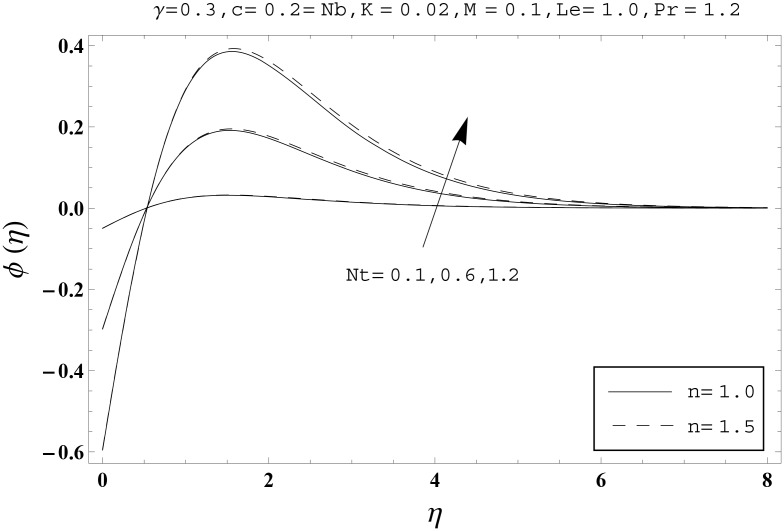
Effects of *n* and *Nt* on *ϕ*(*η*).

Effects of couple stress parameter *K*, magnetic number *M*, ratio parameter *c*, Biot number *γ*, Lewis number *Le*, Prandtl number *Pr*, Brownian motion parameter *Nb* and thermophoresis parameter *Nt* on the dimensionless temperature *θ*(*η*) and nanoparticles concentration *ϕ*(*η*) are sketched in the Figs 3–16. The results are obtained for two different values of *n* just to compare the corresponding profiles in cases of linear and nonlinear stretching surfaces. Fig 3 presents the temperature profiles corresponding to different values of couple stress parameter *K*. Temperature profiles are enhanced for the larger values of couple stress parameter. Moreover the temperature profile is weaker at *n* = 1.0 than at *n* = 1.5. Fig 4 depicts the temperature profiles for various values of magnetic number *M*. Here *M* ≠ 0 corresponds to the hydromagnetic flow case and *M* = 0 is for the hydrodynamic flow situation. We examined that the temperature profiles are higher for hydromagnetic flow in comparison to the hydrodynamic flow. An increase in the values of magnetic number causes stronger temperature profiles. Moreover the thermal boundary layer thickness is lower in linear stretching surface case (*n* = 1.0) when compared with the nonlinear stretching surface case (*n* = 1.5). Fig 5 shows the temperature profiles for different values of ratio parameter *c*. Larger values of ratio parameter creates a reduction in the temperature profiles and related thermal boundary layer thicknesses. For *c* = 0, the two-dimensional flow situation is achieved. It is also noticed that the temperature profiles for two-dimensional flow are lower when compared with the three-dimensional situation. Fig 6 presents the temperature profiles for various values of the Biot number *γ*. Here the temperature profiles are enhanced when we increase the values of Biot number. An increase in the Biot number *γ* causes stronger convection which shows higher temperature profiles and more thermal boundary layer thicknesses. It is also seen that the temperature profiles show similar behavior for both linear and nonlinear stretching surfaces. Fig 7 depicts that the temperature profiles are lower for the larger values of Prandtl number *Pr*. Prandtl number has an inverse relationship with the thermal diffusivity. An enhancement in the Prandtl number causes a weaker thermal diffusivity. Such weaker thermal diffusivity corresponds to lower temperature profiles and thermal boundary layer thicknesses. Moreover the thermal boundary layer thickness is less for linear stretching surface (*n* = 1.0) in comparison to the nonlinear stretching surface (*n* = 1.5). Fig 8 shows the temperature profiles for different values of thermophoresis parameter *Nt*. Temperature profiles are enhanced when we increase the values of thermophoresis parameter. Larger values of *Nt* causes a stronger thermophoresis force which tends to move nanoparticles from hot to cold region and as a result the temperature profile enhances. It is also observed that the thermal boundary layer thicknesses show similar behavior for both linear and nonlinear stretching surfaces. Fig 9 presents the nanoparticles concentration profiles corresponding to various values of couple stress parameter *K*. Larger values of couple stress parameter leads to stronger nanoparticles concentration distributions and more nanoparticles concentration boundary layer thicknesses. It is also shown that the nanoparticles concentration field is higher in nonlinear stretching surface case (*n* = 1.5) when compared with the linear stretching surface (*n* = 1.0). Fig 10 depicts that the nanoparticles concentration profiles are enhanced when we increase the values of magnetic number *M*. Moreover the nanoparticles concentration profiles show similar behavior for both linear and nonlinear stretching surfaces. Fig 11 shows the nanoparticles concentration profiles corresponding to different values of the ratio parameter *c*. Here the nanoparticles concentration profiles are lower for the larger values of ratio parameter. It is also noticed that the nanoparticles concentration profiles show decreasing behavior for both linear and nonlinear stretching surfaces. Effects of Biot number *γ* on the nanoparticles concentration distributions are presented in the Fig 12. Nanoparticles concentration profiles are higher for the larger values of Biot number. Moreover the nanoparticles concentration profiles show similar behavior for both linear and nonlinear stretching surfaces. Fig 13 presents that the nanoparticles concentration profiles are lower for the larger values of Lewis number *Le*. Lewis number has an inverse relationship with the Brownian diffusion coefficient. An enhancement in the Lewis number leads to a lower Brownian diffusion coefficient. Such lower Brownian diffusion coefficient causes weaker nanoparticles concentration profiles. It is also observed that the nanoparticles concentration distributions show decreasing behavior for both linear and nonlinear stretching surfaces. Effects of Prandtl number *Pr* on the nanoparticles concentration profiles are sketched in Fig 14. We observed that an enhancement in the Prandtl number produces weaker nanoparticles concentration profiles. Moreover the nanoparticles concentration profiles depict similar behavior for both linear and nonlinear stretching surfaces. Fig 15 shows that the larger values of Brownian motion parameter *Nb* creates lower nanoparticles concentration profiles. It is also seen that the nanoparticles concentration distributions are lower for both linear and nonlinear stretching surfaces. Effects of thermophoresis parameter *Nt* on the nanoparticles concentration profiles are presented in Fig 16. It is clearly observed that the nanoparticles concentration profiles are enhanced for the larger values of thermophoresis parameter. Moreover the nanoparticles concentration field is weaker for linear stretching surface (*n* = 1.0) when compared with the nonlinear stretching surface (*n* = 1.5). Table 2 depicts the values of skin-friction coefficients $-{Re}_{x}^{1/2}C_{fx}$ and $-{Re}_{y}^{1/2}C_{fy}$ for various values of power-law index *n*, couple stress parameter *K*, magnetic number *M* and ratio parameter *c*. Here the skin-friction coefficients are increasing functions of power-law index *n*. It is also seen that the skin-friction coefficients are enhanced for the larger values of magnetic number *M*. Values of local Nusselt number ${Re}_{x}^{-1/2}{Nu}_{x}$ for various values of *n*, *K*, *M*, *c*, *γ*, *Nt*, *Nb*, *Le* and *Pr* are computed in Table 3. We noticed that the larger values of couple stress parameter *K*, magnetic number *M* and thermophoresis parameter *Nt* correspond to a lower local Nusselt number while opposite behavior is observed for Biot number *γ*. Table 4 presents the values of temperature and nanoparticles concentration profiles for various values of *n*, *K*, *M*, *c*, *γ*, *Nt*, *Nb*, *Le* and *Pr* when *η* = 1.0. From this Table, it is clearly shown that the Biot number *γ* and Prandtl number *Pr* influences the temperature and nanoparticles concentration profiles the most.

**Table 2:** Values of skin-friction coefficients $-{Re}_{x}^{1/2}C_{fx}$ and $-{Re}_{y}^{1/2}C_{fy}$ for various values of *n*, *K*, *M* and *c*.

*K*	*M*	*c*	$-{Re}_{x}^{1/2}C_{fx}$		$-{Re}_{y}^{1/2}C_{fy}$	
			*n* = 1.0	*n* = 1.5	*n* = 1.0	*n* = 1.5
0.00	0.1	0.2	1.1000	1.3050	2.4597	2.9181
0.01			1.0932	1.2945	2.4446	2.8945
0.02			1.0863	1.2832	2.4290	2.8694
0.02	0.0	0.2	1.0819	1.2786	2.4192	2.8591
	0.2		1.0993	1.2970	2.4581	2.9001
	0.5		1.1860	1.3886	2.6520	3.1050
0.02	0.1	0.2	1.0863	1.2832	2.4290	2.8694
		0.3	1.1290	1.3332	2.0613	2.4341
		0.5	1.2095	1.4270	1.7105	2.0181

**Table 3:** Values of local Nusselt number ${Re}_{x}^{-1/2}{Nu}_{x}$ for various values of *n*, *K*, *M*, *c*, *γ*, *Nt*, *Nb*, *Le* and *Pr*.

*K*	*M*	*c*	*γ*	*Nt*	*Nb*	*Le*	*Pr*	${Re}_{x}^{-1/2}{Nu}_{x}$	
								n = 1.0	n = 1.5
0.00	0.1	0.2	0.3	0.1	0.2	1.0	1.2	0.2114	0.2356
0.02								0.2112	0.2352
0.05								0.2108	0.2344
0.02	0.0	0.2	0.3	0.1	0.2	1.0	1.2	0.2113	0.2353
	0.5							0.2091	0.2329
	0.8							0.2060	0.2295
0.02	0.1	0.0	0.3	0.1	0.2	1.0	1.2	0.2054	0.2287
		0.3						0.2136	0.2379
		0.5						0.2180	0.2427
0.02	0.1	0.2	0.1	0.1	0.2	1.0	1.2	0.0877	0.0979
			0.7					0.3527	0.3917
			1.2					0.4458	0.4943
0.02	0.1	0.2	0.3	0.0	0.2	1.0	1.2	0.2115	0.2355
				0.5				0.2100	0.2338
				1.0				0.2084	0.2321
0.02	0.1	0.2	0.3	0.1	0.5	1.0	1.2	0.2112	0.2352
					1.0			0.2112	0.2352
					1.5			0.2112	0.2352
0.02	0.1	0.2	0.3	0.1	0.2	0.5	1.2	0.2113	0.2353
						1.0		0.2112	0.2352
						1.5		0.2111	0.2351
0.02	0.1	0.2	0.3	0.1	0.2	1.0	0.5	0.1687	0.1874
							1.0	0.2034	0.2263
							1.5	0.2200	0.2451

**Table 4:** Values of temperature and nanoparticles concentration profiles for various values of *n*, *K*, *M*, *c*, *γ*, *Nt*, *Nb*, *Le* and *Pr* when *η* = 1.0.

*K*	*M*	*c*	*γ*	*Nt*	*Nb*	*Le*	*Pr*	Temperature		Concentration	
								*n* = 1.0	*n* = 1.5	*n* = 1.0	*n* = 1.5
0.1	0.1	0.2	0.3	0.1	0.2	1.0	1.2	0.12041	0.12292	0.01230	0.01133
0.3								0.12673	0.13022	0.00970	0.00823
0.5								0.13436	0.13885	0.00643	0.00447
0.02	0.1	0.2	0.3	0.1	0.2	1.0	1.2	0.11826	0.12038	0.01316	0.01238
	0.3							0.11989	0.12191	0.01257	0.01184
	0.5							0.12296	0.12479	0.01147	0.01084
0.02	0.1	0.1	0.3	0.1	0.2	1.0	1.2	0.12676	0.12867	0.01000	0.00928
		0.3						0.11035	0.11264	0.01605	0.01523
		0.5						0.09619	0.09871	0.02105	0.02022
0.02	0.1	0.2	0.1	0.1	0.2	1.0	1.2	0.04879	0.04969	0.00511	0.00478
			0.3					0.11826	0.12038	0.01316	0.01238
			0.5					0.16523	0.16810	0.01913	0.01807
0.02	0.1	0.2	0.3	0.1	0.2	1.0	1.2	0.11826	0.12038	0.01316	0.01238
				0.3				0.11927	0.12139	0.03941	0.03708
				0.5				0.12029	0.12241	0.06557	0.06167
0.02	0.1	0.2	0.3	0.1	0.1	1.0	1.2	0.11826	0.12038	0.02632	0.02476
					0.3			0.11826	0.12038	0.00877	0.00825
					0.5			0.11826	0.12038	0.00526	0.00495
0.02	0.1	0.2	0.3	0.1	0.2	0.1	1.2	0.11810	0.12022	-0.03558	-0.03568
						0.3		0.11813	0.12025	-0.02244	-0.02272
						0.5		0.11816	0.12028	-0.01056	-0.01101
0.02	0.1	0.2	0.3	0.1	0.2	1.0	0.1	0.25066	0.25086	-0.03989	-0.03997
							0.3	0.22398	0.22457	-0.02871	-0.02893
							0.5	0.19834	0.19930	-0.01815	-0.01851

## Main findings

Three-dimensional flow of couple stress nanofluid over a nonlinear stretching surface with the convective surface boundary condition and non-uniform magnetic field is analyzed. The main findings of the present research are given below:

- An increase in the couple stress parameter *K* shows an enhancement in the temperature and nanoparticles concentration profiles.
- Temperature and nanoparticles concentration profiles are enhanced with an increase in the values of magnetic number *M*.
- Effects of Biot number *γ* on the temperature and nanoparticles concentration profiles are qualitatively similar.
- Temperature *θ*(*η*) and thickness of the thermal boundary layer are lower for the larger values of Prandtl number *Pr*.
- Larger values of thermophoresis parameter *Nt* present similar behavior for temperature and nanoparticles concentration profiles.
- An enhancement in the Brownian motion parameter corresponds a weaker nanoparticles concentration profile.
- Coefficients of skin-friction are higher for the larger values of the magnetic number *M*.
- Rate of heat transfer at the wall is constant for Brownian motion parameter while it is lower for the thermophoresis parameter.
